# Biomathematical pattern of EMG signal propagation in smooth muscle of the non-pregnant porcine uterus

**DOI:** 10.1371/journal.pone.0173452

**Published:** 2017-03-10

**Authors:** Malgorzata Domino, Bartosz Pawlinski, Zdzislaw Gajewski

**Affiliations:** Department of Large Animal Diseases with Clinic, Veterinary Research Centre and Center for Biomedical Research, Faculty of Veterinary Medicine, Warsaw University of Life Sciences (WULS – SGGW), Warsaw, Poland; UNITED STATES

## Abstract

Uterine contractions are generated by myometrial smooth muscle cells (SMCs) that comprise most of the myometrial layer of the uterine wall. Aberrant uterine motility (i.e., hypo- or hyper-contractility or asynchronous contractions) has been implicated in the pathogenesis of infertility due to the failure of implantation, endometriosis and abnormal estrous cycles. The mechanism whereby the non-pregnant uterus initiates spontaneous contractions remains poorly understood. The aim of the present study was to employ linear synchronization measures for analyzing the pattern of EMG signal propagation (direction and speed) in smooth muscles of the non-pregnant porcine uterus *in vivo* using telemetry recording system. It has been revealed that the EMG signal conduction in the uterine wall of the non-pregnant sow does not occur at random but it rather exhibits specific directions and speed. All detectable EMG signals moved along the uterine horn in both cervico-tubal and tubo-cervical directions. The signal migration speed could be divided into the three main types or categories: i. slow basic migration rhythm (SBMR); ii. rapid basic migration rhythm (RBMR); and iii. rapid accessory migration rhythm (RAMR). In conclusion, the EMG signal propagation in smooth muscles of the porcine uterus *in vivo* can be assessed using a linear synchronization model. Physiological pattern of the uterine contractile activity determined in this study provides a basis for future investigations of normal and pathologicall myogenic function of the uterus.

## Introduction

Uterine contractions are generated by myometrial smooth muscle cells (SMCs) comprising most of the myometrial layer of the uterine wall. Synchronous contractions of SMCs are responsible for normal gamete transport in the female reproductive tract and contribute to the expulsion of uterine debris [1]. The occurrence of uterine contractions must be strictly controlled and coordinated to sustain these reproductive functions [1, 2]. Abnormal patterns of uterine motility such as hypoactive, hyperactive or asynchronous contractions have been implicated in the pathogenesis of infertility, implantation failure, endometriosis and abnormal estrous cycles [3]. However, the specific mechanism by which the non-pregnant uterus autonomously initiates myometrial contractions remains poorly described. In spite of their clinical significance, contractile properties of the uterus during the entire estrous cycle have not been examined [4]. A better understanding of the complex myometrial activity would help to develop effective therapies for an array of reproductive disorders associated with the flawed rhythmicity of uterine contractions.

A single impulse can initiate a myometrial contraction but multiple, coordinated impulses are needed for forceful and sustained contractions [5]. Moreover, individual impulses within the myometrial tissue may differ in speed and direction, and such intrinsic variations in conduction velocities are essential for sustaining forceful and coordinated muscular contractions [6, 7]. Uterine SMCs themselves trigger cellular processes resulting in uterine contractions. The typical sequence of these interrelated processes is referred to as the excitation-contraction (EC) coupling [8] and entails three stages: Ca^2+^ entry into the cell; Ca^2+^ expulsion from the cell; and SMC contraction. Calcium influx occurs through the voltage-dependent calcium channels in response to depolarization of the cell membrane and results in a transient increase in intracellular concentrations of Ca^2+^ ions [1, 2]. The voltage- and time-dependent changes in the membrane ionic permeability initiate an action potential (AP) or myoelectrical activity [5]. Cell membrane stimulated by external impulses responds with a change in the transmembrane electrical potential, which leads to opening of membrane channels to let ions enter or leave the cell [8]. Calcium ions constitute the main component of depolarizing current during the propagation of AP as well as the most important factor determining contractile force of myometrial myocytes [1]. Elevated level of intracellular Ca^2+^ ions allows for binding of Ca^2+^ by calmodulin, which ultimately results in activation of the light-chain myosin kinase and phosphorylation. Subsequently, the cross-bridges between actin and myosin filaments are formed and SMCs contract [1, 2]. There is a paucity of studies on the generation of EC processes by the uterine SMCs *in situ*. Such investigations are confined to the determination of intrauterine pressure and uterine electrical activity during simulated uterine contractions [8, 9]. For example, Cochran and Gao [9] designed the electromechanical model to simulate intrauterine pressure and to assess uterine contractions during parturition. Sharifimajd et al. [8] developed an electro-chemo-mechanical model to replicate excitation, activation, and contraction of uterine smooth muscle cells. In both cases, uterine contractions were induced “from cellular level to the organ level” due to electrophysiological properties of the pacemaker cells and ensuing propagation of electrical discharges via gap junctions within the myometrium. Most of earlier studies on uterine activity deal primarily with the electrophysiology of the SMC membrane [4, 10]. In all *in vitro* investigations, at the level of single myometrial myocyte, equations describing various ionic membrane currents were established from their respective activation curves. Parameters were determined using the voltage-clamp technique and measurements performed on isolated cells [11]. This particular approach is excellent for describing contribution of cellular phenomena in muscular contractions but provides inadequate information on contractile activity of the tissue or organ [8, 12]. Alternatively, *in vivo* models of uterine contractility utilize the “top-down approach” in which function in the entire organ or tissue following activation of SMCs is monitored.

Changes in electrical activity of smooth muscle fibers during contraction or relaxation can be detected on surface of skin (surface EMG) or directly within the myometrium (needle EMG) as an electromyography (EMG) signal. The EMG is the sum of bioelectrical signals or electric currents produced by differences in bioelectrical potentials along a specialized tissue and is associated with electrochemical events occurring during the propagation of action potential [13]. Two distinctive forms of action potential have been recorded in myometrium of various species, namely a single action potentials and multiple action potentials. The single action potential, called the "single spike", consists of rapid depolarization followed by immediate repolarization of cell membranes. These spikes often occur in sequels called "bursts", number and frequency of spikes within burst predetermine strength and rapidness of smooth muscle contraction. Multiple spontaneous action potentials (bursts) seem to arise simultaneously in different regions of the uterine wall [1, 12]. The frequency and duration of the bursts as well as the frequency of spikes within a burst shows considerable individual variations and vary with the stage of parturition [7]. The propagation of electrical activity in the myometrium mainly depends on arrangement of myometrial bundle fibers [6], population of gap junctions formed between adjacent cells during each contraction [14, 15], and waves of calcium transport [16]. In contrast to skeletal muscle, in which the propagation of action potentials that progresses in muscle fibers is highly predictable, the direction and speed of electrical activation in myometrial cells remain to be elucidated [17]. Most of previously published studies were devoted to the analysis of entire bursts or single spikes in pregnant uterus [8, 18, 19, 20], and only few studies dealt with non-pregnant state [4, 21]. Hence, the main goal of the present study was to determine the biomathematical pattern describing EMG signal propagation in the non-gravid porcine uterus *in vivo*.

## Materials and methods

### Data acquisition

**Fig 1:**
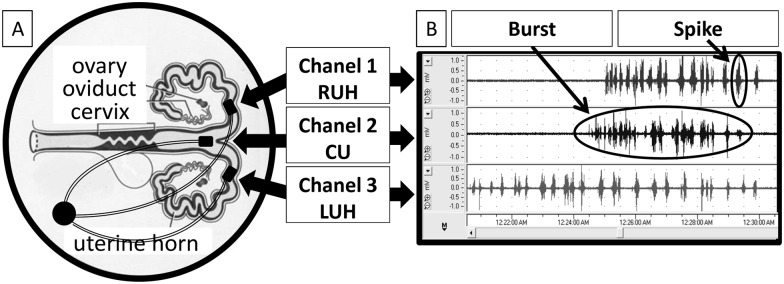
Diagram of the measurement system. Electrodes were arranged in the porcine reproductive tract (A) and the EMG signals (B) were sampled from: channel 1, right uterine horn, RUH; channel 2, corpus uteri, CU; and channel 3, left uterine horn, LUH.

The experiment has been conducted on 10 mature Polish Landrace sows (n = 10). The experiment has been conducted according to applicable national and international ethical guidelines and all efforts were made to minimize animals suffering. The protocol was approved by the III Local Ethical Committee on Animal Testing in Warsaw (Permit Number: 71/2009, from 19.11.2009) on behalf of the National Ethical Committees on Animal Testing. Sows had been adapted to the animal facilities for 7 days before studies. During the entire experiment animals were housed in metabolic cages, fed and watered *ad libitum*. In order to maintain animal welfare surgery was carried out under general anesthesia and the telemetry EMG recording method was applied. Telemetry method [22] allows to reduce the number of animals used in the experiment and provide long-term registration without stress connected with immobilization. Spontaneous uterine activity in non-pregnant state during diestrus was recorded by the combination of three electrodes connected to 3-channel transmitter used in large animals. The telemetry transmitter TL10M3-D70-EEE (DSI, St. Paul, Minnesota, USA) was surgically positioned between abdominal muscles and three silver bipolar needle electrodes were sutured onto different topographic regions of uterus: right uterine horn (RUH—channel 1), corpus uteri (CU—channel 2) and the left uterine horn (LUH—channel 3) surfaces (Fig 1). The distance between electrodes (channel 1 to channel 2 and channel 2 to channel 3) was constant and fixed at 17 cm. Animals were premedicated with an intramuscular injection of azaperone (Stresnil, 3 [mg/kg b.wt.], i.m., Janssen Pharmaceutica) and then catheter was inserted into the auricular vein. Surgery was carried out under general anesthesia, consisting of combined administration of medetomidyne (Cepetor, 1 [mg/kg b.wt.], i.v., CP- Pharma Handelsges), butorphanol (Butomidor, 0,2 [mg/kgb.wt.], i.v., Ricgter Pharma AG), ketamine (Bioketan, 3 [mg/kg b.wt.], i.v., Vetoquinol Biowet) and propofol (Propofol, 2–4 [mg/kg b.wt.], i.v., Pfizer). Then pigs recovered from surgery analgesic—meloxicam (Metacam 0.4 [mg/kg b.wt.], i.m., Boehringer Ingelheim) and anti-microbial—cefquinom (Cobactan, 2.0 [mg/kg b.wt.], i.m., Intervet) had been administered for 5 days. Obtained analog signal was digitalized and sent by radio waves to the telemetric receiver (DL10 analog output (DSI)). The signal was acquired with a 3-channel transmitter (PowerLab (ADInstruments, Melbourne, Australia) and analyzed. Sampling frequency was 100 Hz. Pigs were euthanized at the end of the experiment by—Sodium Pentobarbital (Morbital 100.0 or [mg/kg b.wt.], i.v., Biowet Pulawy).

### EMG data processing

The EMG signals were digitally filtered with a band-pass filter [5–50 Hz], then power line interference was lowered from the EMG recordings with a notch filter. Mean and linear trends were removed [23].

Uterine contractions were defined as series of electrical potentials with amplitude above 5 μV and a duration of more than 3 s, separated from each of next series by a time period not less than 5 s. Any new electrical activity after this period (5 s) was interpreted as a subsequent contraction (8). Each contraction was described using spike and burst parameters. The spike represented single action potentials while the burst comprised multiple action potentials. Mean amplitude [mV], mean RMS (root mean square) [mV], duration of electrical activity [s], duration of pauses [s], and number of spikes forming a burst were analyzed relative to bursts and spikes, respectively.

The EMG signal spectral content was analyzed in addition to studying changes in its time domain features. The spectrum analysis gave amplitudes of each of pure tones of different frequencies and phases, summed in single EMG signal. The amplitude spectrum of investigated signals was defined as the distribution of amplitudes over different frequencies. By means of Fourier analysis (FFT—Fast Fourier transform), dominant frequency (DF) [Hz] (the frequency at which most signal energy was transmitted) was assessed for each data series. We used the Hamming window only for the Fourier analysis [21, 24].

### EMG channel synchronization

The uterus is indubitably a complex system in which billions of cells comprising myometrium interact in a complex manner. Our understanding of the co-ordination of uterine contractions is incomplete. In physiological research, multivariate data sets containing two or more simultaneously recorded time series are usually examined to establish signals similarity [24]. We used similarity measures based on the concept of time series data synchronization. In order to describe similarities between EMG signals, we used two-dimensional functions analysis. Degree of synchronization between three simultaneously recorded data series (channels 1(x), 2(y), 3(z)) was estimated for two signal pairs (xy and yz) using linear measures: the cross-correlation function (ƒ_x,y_(l), ƒ_y,z_(l)) and the cross-coherence function (C_xy_(ƒ), C_yz_(ƒ)). The coherence function in frequency domain was equivalent to the correlation function in time domain. C_xy_ was estimated after Fourier analysis (FFT) [21].

#### Cross-correlation analysis

Cross-correlation function gives the correlation degree between the two signals—amplitude data series (pair xy—channel 1 and channel 2 / pair yz—channel 2 and channel 3) [25]. The cross-correlation function of two discrete data series x(n) and y(n) is a statistical quantity defined as follows:

$$ƒx,y\left(l\right)\;=\frac{cov\left(x,y\right)}{Sd_{x}x\;Sd_{y}}$$ (1)

Signals x(n), y(n) and z(n) are collected from the right uterine horn, corpus uteri and left uterine horn electrodes, respectively.

After normalization, the value of cross-correlation parameters will be between -1 and 1. The result is 1 if x(n) = y(n) or y(n) = z(n) and l = 0; *ƒx*,*y(l)* = 0 if x,y are statistically independent and it is -1 if x(n) = —y(n) or y(n) = —z(n) and l = 0. Synchronization is high if |*ƒx*,*y(l)*| = 1 and absent if *ƒx*,*y(l)* = 0.

#### Cross-coherence analysis

The cross-coherence function gives the coherence degree between two signals—dominant frequencies data series (pair xy—channel 1 and channel 2 / pair yz—channel 2 and channel 3) [25]. The cross-coherence function of two discrete data series x(n) and y(n) is a statistical quantity defined as follows:

$$C_{xy}\left(ƒ\right)=\frac{{\left|P_{xy}\left(ƒ\right)\right|}^{2}}{P_{xx}\left(ƒ\right)\;x\;P_{yy}\left(ƒ\right)}$$ (2)

It is real-valued, positive and normalized to vary between 0 (no coherence) and 1 (complete coherence). In the same way, high synchronization corresponds to a value of 1, whereas a value of 0 indicates lack of synchronization.

#### Description of the experimental model

Values for the cross-correlation coefficient |r| and cross-coherence coefficient |R| were estimated separately for two pairs of discrete time series x(n) and y(n) as well as y(n) and z(n). Synchronization between the right uterine horn (x(n)) and corpus uteri (y(n)) as well as the corpus uteri (y(n)) and left uterine horn (z(n)) was observed. The threshold at which the values of |r| and |R| were considered significant was determined empirically, and was always over > 0.50 with significant strength from middle to very high. The burst pairs with an empirical value of |R| over threshold were considered as synchronized. Results of linear synchronization measures application to pairs of EMG signals demonstrated that the cross-coherence function is effective in determining bursts similarity. Similarity measures based on the concept of synchronization allow detection of bursts propagation in different topographic regions. They were used to "identify" the frequency fingerprint of the spatial burst and determined when the burst reached the other location during propagation.

Locations of the first and second electrodes (on the x/y or y/z axis) were used to measure highly synchronized signals, and the direction of bursts propagation was evaluated. The second signal location (on the x/y or y/z axis) in the time function pointed towards cervico-tubal and tubo-cervical directions.

The speed of bursts propagation was evaluated based on the time elapsed for highly synchronized signals to move from the first electrode to the second one (measuring points on the x/y or y/z axis).

#### Statistical analysis

To assess statistical differences among three channel data series, a one-way ANOVA and the Kruskal-Wallis test (the level of statistical significance was set to P<0.05) were performed. To evaluate the percentage of signal propagation (direction and speed) in relation to the total number of synchronizations examined, non-parametric, two-tailed Mann-Whitney test was applied (P<0.01). The independence of speed was tested using a non-parametric Kruskal-Wallis test with Dunn's multiple comparisons test (P<0.05) afterwards, while the homogeneity of speed populations was defined using a one-way ANOVA test followed by Tukey's multiple comparisons test (P<0.05) using GraphPad Prism 6 (GraphPad Software Inc., San Diego, CA, USA).

## Results

Successful EMG recordings were obtained from each electrode site. Over 240 bursts were extracted from each animal during long time measurement (>24 hours of continuous recording). From all registered bursts only those with high similarity were used as a “fingerprint” to establish the direction of propagation and propagation speed. The characteristic for porcine uterus myoelectrical activity pattern was established based on burst parameters (Table 1). There were no significant differences in the mean amplitude, RMS and duration between different topographic regions (P>0.05).

**Table 1:** EMG signals parameters (mean % ± SEM) of bursts in different topographic regions of uterus.

Topographic regions	EMG parameters		mean	SEM
**Right uterine horn**	**A**^a^	[mV]	0.74	±0.021
	**RMS**^b^	[mV]	0.10	±0.001
	**T**^c^	[s]	221.60	± 25.531
**Corpus uteri**	**A**^a^	[mV]	0.57	± 0.021
	**RMS**^b^	[mV]	0.07	± 0,001
	**T**^c^	[s]	209.70	± 22.920
**Left uterine horn**	**A**^a^	[mV]	1.18	± 0.052
	**RMS**^b^	[mV]	0.15	± 0.003
	**T**^c^	[s]	195.20	± 20.522

^a^ A—amplitude,

^b^ RMS—root mean square,

^c^ T—time—duration of burst.

**Fig 2:**
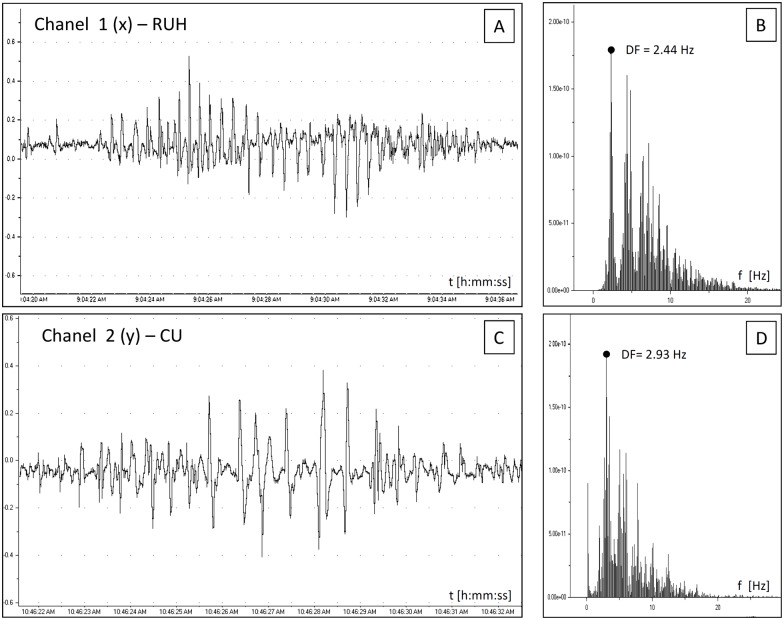
Fourier analysis of highly synchronized EMG signals. EMG signals from the right uterine horn (A, B) and corpus uteri (C, D) in time (A, C) and frequency (B, D) domains.

**Fig 3:**
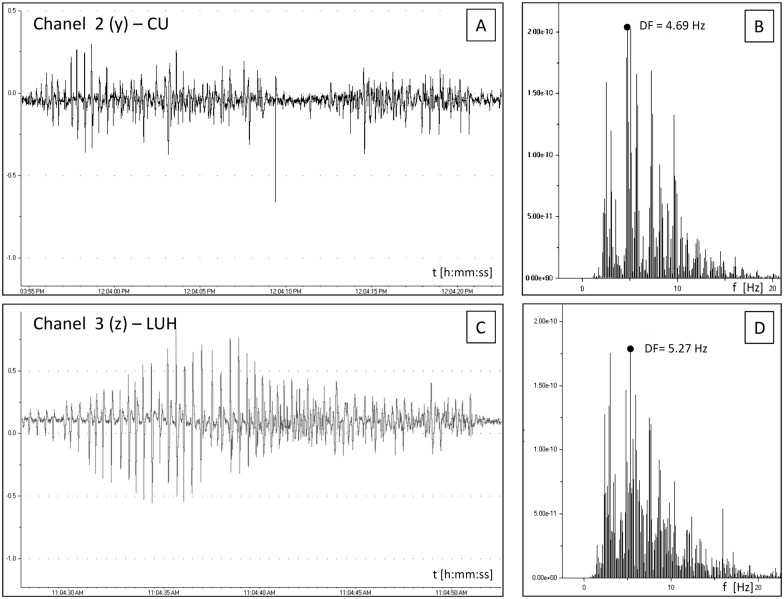
Fourier analysis of highly synchronized EMG signals. EMG signals from the corpus uteri (A, B) and left uterine horn (C, D) in time (A, C) and frequency (B, D) domains.

There were no significant differences in the mean |R| (P = 0.42) and |r| (P = 0.30) values between the right (x(n) and y(n) discrete data series) and left uterine horn (y(n) and z(n) discrete data series. The mean (±SEM) values of similarity measures were (|r| = 0.22±0.02; {0.08 |r| Є R: 0.50 ≤ |r| ≤ 1}) and (|R| = 0.23±0.02; {0.17 |R| Є R: 0.50 ≤ |R| ≤ 1}) for the cross-correlation and cross-coherence, respectively. The cross-coherence function demonstrated a significantly higher similarity rate and was used to determine the direction and speed of signal propagation in the complex biological system of uterine myometrium. Figs 2 and 3 demonstrate Fourier analysis of highly synchronized uterine contraction signals in different topographic regions: RUH and CU (Fig 2) as well as CU and LUH (Fig 3). Values of DFs, which are marked by black dots, are very similar for these two signal pairs. Highly synchronized time series (empirically considered |R|>0.50) are built and details components shown in Fig 1. They were used to determinate the direction and speed of signal propagation.

The EMG signals were propagated along the uterine horn in both cervico-tubal (from CU to UH) and tubo-cervical (from UH to CU) directions. There were no significant differences (P = 0.20) between the percentages (mean % ± the standard error of mean (SEM)) of bursts propagated in both directions, from CU to UH (45.30% ± 3.27) and from UH to CU (4.73% ± 3.25) in relation to the total number of highly synchronized uterine contraction signals. There were also no significant differences (P = 0.20 and P = 0.60, respectively) between bursts propagation in the right and left uterine horns (Table 2).

**Table 2:** The percentage (mean % ± SEM) of bursts propagation directions along the uterus in relation to the total number of highly synchronized uterine contraction signals.

The direction	Tubo-cervical	Cervico-tubal
**Right uterine horn**	**RUH-CU** ^a^	**CU-RUH** ^b^
mean [%]	24.5	20.8
SEM	±2.27	±3.75
**Left uterine horn**	**LUH-CU** ^c^	**CU-LUH** ^d^
mean [%]	30.2	24.5
SEM	±1.07	±0.53
**P**	0.20	0.60

^a^ RUH-CU—tubo-cervical direction in the right uterine horn,

^b^ CU-RUH—cervico-tubal direction in the right uterine horn,

^c^ LUH-CU—tubo-cervical direction in the left uterine horn,

^d^ CU-LUH—cervico-tubal direction in the left uterine horn. Mann-Whitney test (P<0.01)

EMG signals were propagated along the uterine horn with three significantly different (P<0.0001), independent speeds: SBMR (slow basic migration rhythm), RBMR (rapid basic migration rhythm) and RAMR (rapid accessory migration rhythm). Bursts propagation speed was determined experimentally and tested for homogeneity inside the population for: SBMR, RBMR and RAMR. All propagation speeds were considered to be homogeneous (P>0.05). The SBMR, RBMR and RAMR values (mean ± SEM) as well as the percentage (mean %) of bursts propagation speed in relation to total number of highly synchronized uterine contraction signals are presented in Table 3.

**Table 3:** The values (mean ± SEM) and the percentage (mean %) of bursts propagation speed along the uterus in relation to the total number of highly synchronized uterine contraction signals.

The speed	SBMR ^a^	RBMR ^b^	RAMR ^c^
mean [mm/min]	1.25	2.47	7.07
SEM	±0.04	±0.08	±0.60
mean [%]	46.01	26.30	27.30

^a^ SBMR—slow basic migration rhythm,

^b^ RBMR—rapid basic migration rhythm,

^c^ RAMR—rapid accessory migration rhythm. For independence: Kruskal-Wallis test with Dunn's multiple comparisons test (P<0.05). For homogeneity: one-way ANOVA test with Tukey's multiple comparisons test (P<0.05).

## Discussion

Changes in the number of spikes affected the amplitude measures in time domain without altering the frequency domain and the DF patterns. This could be an explanation for significantly higher similarity rates in the frequency vs. time domain for burst similarity parameters. Variation in number of spikes can be caused by the progressive and reverse recruitment phenomenon where the muscle area affected by spike within burst enlarges progressively [20]. Moreover, contractions demonstrate different propagation parameters, so within the same burst, analysis of single spike can lead to more extensive information compared to the whole burst analysis [7, 20]. Consequently, the combination of simultaneous single spike and burst analyses used in this study significantly reduce inherent variability in the EMG sensitivity in the porcine myometrium.

In a previous study [21] we demonstrated the usefulness of the cross-coherence function in synchronization between uterine horn and corpus uteri for multiple action potentials in short time measurement (<10 hour of continuous recording). In this study the linear synchronization measured in frequency domain was used to determine direction and speed of signal propagation in long time measurement (>24 hours of continuous recording). We stated that determination of detail of signal propagation in short time measurement is unreliable. Recent studies have suggested that both frequency and synchrony of the uterine electrical activity could be potential indicators of predicting uterine contraction [26]. We stated that frequency and synchrony are also suitable for determining direction and speed of those contractions in the non-pregnant uterus. Nevertheless both of these studies [21, 26] and the current one considered cross-coefficients over > 0.50 based on Fourier and synchronization analysis of experimental data. Therefore we stated that similarity measures based on the concept of synchronization analysis can be implemented with fast algorithms and that this method is suitable for real-time applications. The analysis method may be generalized to animal and human data obtained during labor, in pregnancy and non-pregnant state.

The high accuracy of identifying the frequency fingerprint of the spatial burst allowed to determine the signal migration based on three measuring points. Analysis methods, used in the experiment, eased the limits on the ability to interpret data obtained from a limited number of probes in each horn. Langendijk et al. [27] successfully described spontaneous uterine activity around estrus in sows based on open-end catheter technique and one measuring point. Bower and Zerobin et al. [28, 29] established porcine uterine activity including direction of contractility propagation and used for this purpose two measuring points. Gajewski et al. [22, 30] efficiently completed the myoactivity findings based on three measuring points but didn’t carry out biomatematical signal analysis. The myometrial activity detecting methods result in the limited number of probes. All authors recorded myometrial activity directly within the myometrium (EMG) [22, 28, 29, 30, 31] or used intrauterine pressure (IUP) [27]. An alternative method for monitoring the uterine activity based on surface EMG—EHG (electrohysterogram) allowed to application from 3 [32] to 16 [33] and 64 [20] active electrodes located on abdomen skin. The needle EMG picked up the bioelectrical signal directly associated with the muscular activity of myometrium, while EHG collected signal abundant in noise generated between cells and electrodes. Therefore, the electrical activity recorded directly from uterus may provide essential information about uterine activity and permit the prediction of the EHG associated with the contraction. However different dedicated methods for parameter estimation have been applied, from animal *in vitro* experiments (invasive EMG) and human clinical studies (EHG). Limitations during invasive *in vivo* experiments result from hardware and equipment deficiencies. Implantable telemetry systems, designed for monitoring and collecting data from large animals, have disadvantage such as only 3-channel transmitter, limited source of energy and no possibility to implant more than one transmitter in one animal [30, 31].

The uterine activity in non-pregnant sows was recorded via uterine intraluminal pressure measurements (non-surgical technique) [27]. Oviductal and myometrial activity during the periovulatory period in pigs was described using a long-term electromyography combined with the telemetry recording system (invasive method) [22, 30, 31]. Both of those approaches failed to determine the specific direction of waves of uterine contractions. Others have suggested that uterine contractions occur along uterine horns [28, 29] in two opposite directions, tubo-cervical and cervico-tubal, although the tubo-cervical direction appeared to be predominant. In those studies, the myometrial activity was recorded at two different sites in the tips of both uterine horns [28, 29], and it was suggested that the bulk of myometrial activity originated in those regions and was conveyed along uterine horns in tubo-cervical direction [27]. Both ends of the uterine horn (tubal and the cervical) are tentatively anatomical regions with the strong pacemaker activity [22]. Based on our present observations, EMG signals preceding myometrial contractions in the pig’s uterus are propagated relatively uniformly in both cervico-tubal and tubo-cervical directions. This is in agreement with the results of myoelectrical burst analyses in women where both upward and multidirectional propagation patterns have been reported [33, 34]. This “mixed” propagation pattern may serve to facilitate sperm transport along the uterine horns and/or re-distribution of semen throughout the two horns in the case of unilateral deposition of semen [35]. While the cervico-tubal direction of uterine contractions promotes the distribution of semen over the two horns, the tubo-cervical contractions are important for expelling seminal plasma after mating. Therefore, uniform bidirectional contractions of uterus may play a key role in fertilization process; the direction in which uterine contractions are propagated may ultimately influence the rate of sperm transport and conception [36].

Based on the tubular structure of porcine uterus two directions were analyzed: cervico-tubal and tubo-cervical. Extending the discussion of histology structure of the uterus where billions of cells comprising myometrium interact in a complex manner in longitudinal and circular muscle layers the oblong, the spiral path as well as a special path via pacemaker cells network directions have to be considerate. Those complex pathways of signal may be reflected in speed of signal propagation: the slowest signal propagation via spiral path in circular muscle layer, faster via oblong path in longitudinal muscle layer and the fastest via ICLC network.

Having obtained frequency fingerprint identification of high similar bursts we measured the time lag between signals and, the distance between sensors, and calculated the speed. The speed at which an action potential propagates along a muscle fiber or tissue is referred to as propagation (or conduction) speed [17, 37]. Recent studies described the speed of action potential propagation in the muscular layer of isolated uterine strips in animals [18, 36, 38] and whole uterus in women [20]. Once again the analysis method may be generalized to pig, guinea pig, rabbit, cat and human data obtained during *in vivo* and *in vitro* experiments.

As is evident from the analysis of over 240 bursts extracted from each animal, in many occasions the EMG signal started between the sensors. In this case we observed no synchronization between time series data. The unsynchronized signals were not taken under consideration during speed calculation. We suggested the analysis of propagation speed, based on assumption that the signal spreads out linearly and thus it can be detected at different locations after a certain delay [6, 19, 20]. However, there is a great deal of evidence that the linear propagation of single electrical spikes occurs in the uterus and that speed of their propagation can also be measured [18, 19, 20, 29]. In this study, we employed the concept of simultaneous single spike and burst linear analysis in the frequency domain. We suggested to divide the propagation speed uterus into one of three distinctive categories: slow and rapid basic migration rhythm (SBMR, RBMR) and rapid accessory migration rhythm (RAMR).

In circular and longitudinal muscles of the uterus, the single spike is the result of the depolarization phase. The single spike is able to initiate a short contraction, but multiple, higher-frequency, coordinated spikes forming bursts are necessary to trigger forceful and sustained contractions [14]. We speculate, that in the porcine non-pregnant uterus the bungles are propagated across fibers in circular muscles of uterus and along the fibers in longitudinal muscles. We suspect these two propagation pathways to be connected with the two estimated basic migration rhythms: SBMR in circular and RBMR in longitudinal muscles. The conduction of electrical activity in the uterus relies on the cell-to-cell coupling by gap junctions composed of connexin proteins [5]. The grouping of connexins provides channels of low electrical resistance between cells, and thereby furnishes pathways for efficient conduction of action potentials [14]. Circular muscles (in inner layer of the myometrium) contain more gap junctions than longitudinal muscles (the outer layer of the myometrium) per length unit. In the myometrium bioelectrical signal propagation occurs more rapidly in the longitudinal direction then in the circumferential one [17].

Moreover, the presence of interstitial Cajal-like Cells (ICLC) located among SMCs was demonstrated in the non-pregnant human [39] and porcine [40] myometrium. ICLC have been shown to form a network integrating SMCs, nerves and blood vessels. ICLC possess putative bioelectrical properties based on their morphological features such as numerous gap junctions and presence of calcium handling units typical of pacemaker cells [41]. It was suggested that the c-kit activity of ICLC is involved in the spontaneous rhythmic contractions of uterine myocytes. The contraction frequency in longitudinal layer of uterine strips from human can be reduced or inhibited with imatinib (the c-kit/CD117 tyrosine-kinase inhibitor) in a dose-dependent manner [42]. It is attractive to speculate that cellular networks containing ICLC may be responsible for the third accessory migration rhythm detected in the present study, the RAMR. Rapid bursts could be propagated by the ICLC system including longest cellular processes (except for neurons) that form intricate networks between smooth muscle cells.

## Conclusions

In conclusion, the EMG signal propagation in smooth uterine muscles could be determined using linear synchronization measures. We described for the first time the successful cross-correlation function the EMG signal propagation in a long term *in vivo* experiment in the non-gravid porcine uterus. The pattern of the EMG signal propagation is not random but it occurs in an orderly, bidirectional fashion and at distinctive speed. The spontaneous potentials are propagated equally in both directions between both horns and the uterus. Since other studies have shown that the presence of intercellular contacts appears to be controlled by changing estrogen and progesterone levels, further information is needed to elucidate the coordination of contractility during different stages of the estrous cycle. Nevertheless, the analysis of electrical signal propagation in the uterus offers a unique opportunity to understand the mechanisms underlying uterine contractility both in animals and human beings.
